# Ab Initio Static Exchange–Correlation Kernel across Jacob’s Ladder without Functional Derivatives

**DOI:** 10.1021/acs.jctc.2c01180

**Published:** 2023-02-01

**Authors:** Zhandos Moldabekov, Maximilian Böhme, Jan Vorberger, David Blaschke, Tobias Dornheim

**Affiliations:** † Center for Advanced Systems Understanding (CASUS), D-02826Görlitz, Germany; ‡ Helmholtz-Zentrum Dresden-Rossendorf (HZDR), D-01328Dresden, Germany; ¶ Institute of Theoretical Physics, University of Wroclaw, 50-204Wroclaw, Poland

## Abstract

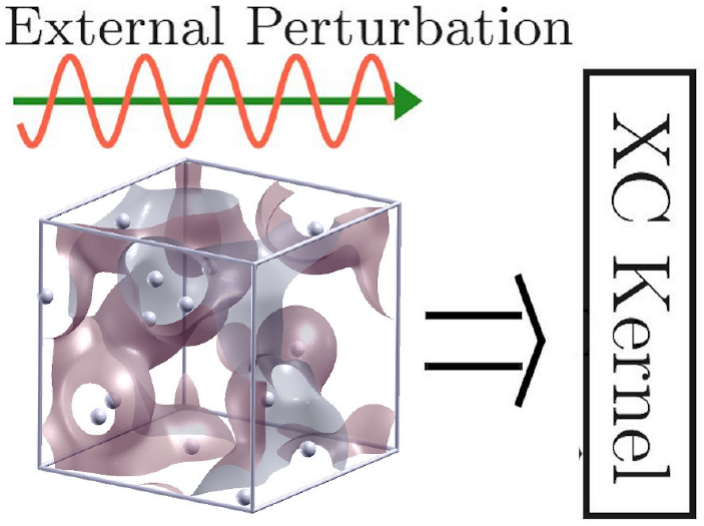

The electronic exchange—correlation (XC) kernel constitutes a fundamental input for the estimation of a gamut of properties such as the dielectric characteristics, the thermal and electrical conductivity, or the response to an external perturbation. In this work, we present a formally exact methodology for the computation of the system specific static XC kernel exclusively within the framework of density functional theory (DFT) and without employing functional derivatives—no external input apart from the usual XC-functional is required. We compare our new results with exact quantum Monte Carlo (QMC) data for the archetypical uniform electron gas model under both ambient and warm dense matter conditions. This gives us unprecedented insights into the performance of different XC functionals, and it has important implications for the development of new functionals that are designed for the application at extreme temperatures. In addition, we obtain new DFT results for the XC kernel of warm dense hydrogen as it occurs in fusion applications and astrophysical objects. The observed excellent agreement to the QMC reference data demonstrates that presented framework is capable to capture nontrivial effects such as XC-induced isotropy breaking in the density response of hydrogen at large wave numbers.

## 1 Introduction

The Kohn–Sham density functional theory (KS-DFT) approach^1,2^ is arguably the most successful simulation tool in many-body physics, quantum chemistry, material science, and several related disciplines. Its main advantage is the evened-out balance between reasonable accuracy and manageable computation cost, which allows for the ab initio description of real materials. Being formally exact,^3^ KS-DFT requires, as external input, the a priori unknown exchange–correlation (XC) functional, which, in practice, must be approximated. Under ambient conditions, when the electrons can usually be assumed to be in their respective ground state, a Jacob’s ladder of functionals^4,5^ serves as a useful categorization of different approximations.

The drastic reduction of computational cost that often renders KS-DFT simulations feasible in the first place is achieved by mapping the original many-electron problem of interest onto an effective single-electron problem. Therefore, DFT gives straightforward access to just the single-electron density *n*_*e*_(**r**) and different contributions to the energy, but two-body (and higher-order) correlation properties are not readily accessible. Thus, an extra challenge emerges of restoring the information about electron–electron correlations needed for the computation of electron structure factors or pair distribution functions. Density functional perturbation theory is a tool to achieve this. In particular, numerous applications such as linear-response time-dependent density functional theory (LR-TDDFT), the computation of electronically screened potentials,^6−8^ quantum hydrodynamics,^9−11^ and the estimation of the energy loss characteristics of high-energy density plasmas^12−14^ require, as an additional input, the system-specific XC-kernel *K*_xc_(**q**, ω) containing higher-order correlations and exchange. For these applications, there had been no possibility to compute the XC kernel for the existing great variety of XC functionals (more than 400) beyond the adiabatic LDA (ALDA) and GGA (AGGA) for extended systems.^15^

**Figure 1:**
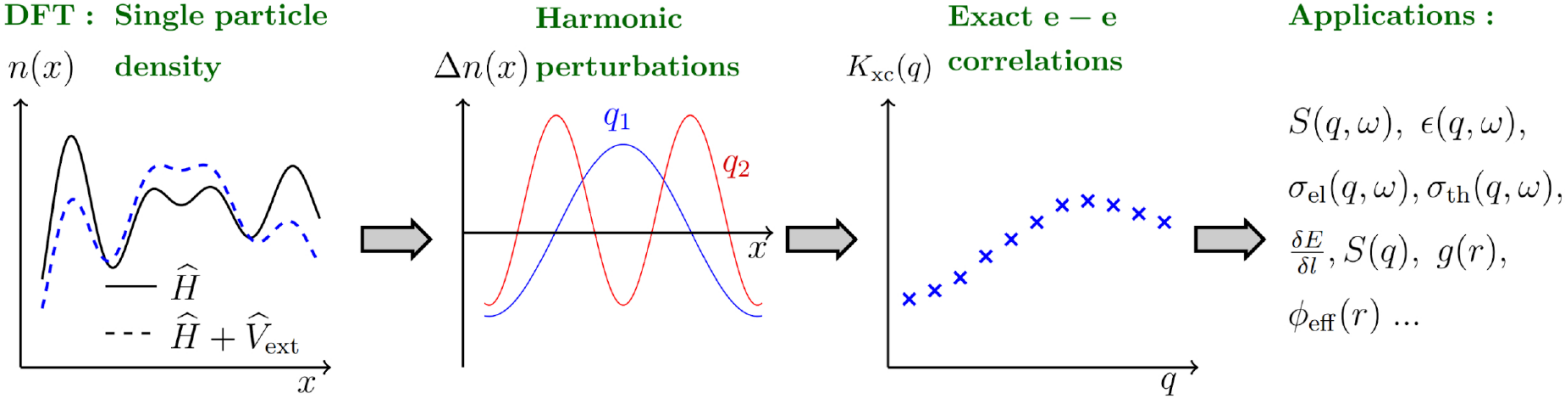
Schematic illustration of the presented methodology. First on the left: We use standard KS-DFT to compute the single-electron density *n*_*e*_(**r**) (a) with respect to the original electronic Hamiltonian of interest  (solid black) and (b) with respect to a modified Hamiltonian subject to an external monochromatic perturbation  (dashed blue). Second from left: We compute the corresponding density modulation Δ*n*_*e*_(**r**) for different wave vectors **q**. Third from left: In the linear-response regime, where , this gives us direct access to the static density response function χ(**q**) and the corresponding XC kernel *K*_xc_(**q**) (blue crosses) *for any XC functional* by inverting eq 4. First on the right: the XC-kernel can be used for the calculation of various electronic properties of the system such as the dynamic structure factor *S*_ee_(**q**, ω), dielectric properties ϵ(**q**, ω), effective potentials ϕ_eff_(*r*), transport properties like electrical σ_*el*_(**q**, ω) and thermal σ_*th*_(**q**, ω) conductivity, and stopping power . Furthermore, the XC-kernel provides access to electron–electron correlation functions such as the static structure factor *S*_*ee*_(**q**), which cannot be readily computed within standard KS-DFT, via the fluctuation–dissipation theorem (FDT).

In this work, we demonstrate a methodology for the ab initio calculation of the static XC kernel—within the framework of KS-DFT—which is fully compatible with the XC potential of self-consistent Kohn–Sham (KS) equilibrium calculations and can be applied for any XC functional. The presented approach completely circumvents the problem of computing functional derivatives, which had been the key obstacle that prevented going beyond AGGA for extended systems. The basic idea is schematically illustrated in Figure 1. In principle, KS-DFT is capable of giving exact results for the single-particle density *n*_*e*_(**r**) for any electronic Hamiltonian  (black curve, leftmost panel). As a second step, the KS-DFT calculation is repeated for a modified Hamiltonian,  =  that is subject to a monochromatic external perturbation of wave vector **q** and perturbation amplitude *A*;^16−20^ this gives one the perturbed single-particle density *n*_*e*_(**r**)_**q**,*A*_ (dashed blue curve, leftmost panel). In combination, direct access to the corresponding density modulation Δ*n*_*e*_(**r**)_**q**,*A*_ = *n*_*e*_(**r**)_**q**,*A*_ – *n*_*e*_(**r**) due to the external perturbation is gained (second panel from left). In the limit of small *A*, when linear response theory^8^ becomes valid, this provides straightforward access to the static linear density response function χ(**q**) = χ(**q**, 0) [cf. eq 3] and, in this way, the static XC kernel *K*_xc_(**q**) = *K*_xc_(**q**, 0) via inverting eq 4 (second panel from right). The latter constitutes the key ingredient to many applications, such as the interpretation of XRTS experiments,^21−25^ the incorporation of electronic XC effects into quantum hydrodynamics,^9−11^ the construction of effective electronically screened potentials,^6−8^ the conductivity,^26^ and ionization potential depression.^27^ Moreover, the fluctuation–dissipation theorem (FDT) [cf. eq 10 below] gives a direct relationship between the thus obtained density response and a correlation function of two density operators, which provides the basis for LR-TDDFT calculations of the dynamic structure factor *S*_ee_(**q**, ω). Consequently, the presented framework for the XC-kernel opens up the enticing possibility to obtain the static structure factor *S*_ee_(**q**)—the Fourier transform of the pair correlation function *g*_ee_(**r**)—of two electrons *exclusively within DFT and without any additional external input* apart from the usual XC functional of standard DFT.

To rigorously demonstrate the correctness and utility of this direct perturbation-based approach, we consider two representative systems. The first example is given by the uniform electron gas (UEG),^8,28,29^ the archetypical electronic system that constitutes the basis for a gamut of applications such as the BCS theory of superconductivity^30^ and Fermi liquid theory.^8^ In the context of the present work, the UEG has the considerable advantage that reliable benchmark data for many properties are available based on highly accurate quantum Monte Carlo (QMC) calculations.^31,32^ As a second, more challenging example, we consider hydrogen, the most abundant element in our universe, which is the subject of active investigation.^33−37^ Indeed, many fundamental questions about hydrogen such as the precise nature and location of the insulator-to-metal phase transition^34^ remain unanswered. Here, we use our new methodology to obtain the static XC kernel of hydrogen and find very good agreement to the recent exact QMC results by Böhme et al.^37^

To illustrate the applicability of our approach across temperature regimes, we consider both the electronic ground state (i.e., the zero-temperature limit, *T* = 0) and highly excited states at the electronic Fermi temperature, Θ = *k*_B_*T*/*E*_F_ = 1 (with *E*_F_ being the usual Fermi energy^8^). In fact, such extreme states are ubiquitous in nature^38^ and occur in astrophysical objects such as giant planet interiors^39^ and brown dwarfs.^40^ Moreover, they are highly relevant for cutting-edge technological applications such as inertial confinement fusion^41^ and the discovery of novel materials.^42−44^ This *warm dense matter* regime is notoriously hard to describe;^45,46^ from the perspective of DFT, one requires a density functional of the XC free energy (*F*_xc_) that explicitly depends on the electronic temperature *T*.^47−49^ While first promising developments^50−53^ have become available over the last years, the field of finite-*T* XC-functionals still remains in its infancy, and the performance of various approximations^54,55^ is substantially less understood, compared to the case of *T* = 0. Indeed, the bulk of DFT calculations for WDM is carried out on the basis of the *zero-temperature approximation* where the actual *T*-dependent XC-free energy is approximated by a suitable ground-state functional.

For the computation of the static XC kernel *K*_xc_(**q**), we use several different *T* = 0 and finite-*T* XC functionals. This gives us unprecedented insights into the performance of different widespread approximations. In practice, we find that ground-state functionals are often more accurate than supposedly more-consistent finite-*T* functionals in the WDM regime; this has profound consequences for the future construction of a new generation of XC functionals that are specifically designed for the application in the WDM regime. Moreover, we show that our new framework is capable to give highly accurate results for the XC kernel, even for the complicated case of partially ionized hydrogen. Therefore, we are convinced that the methodology presented and demonstrated in this work will open up a gamut of avenues for future research, and facilitate unprecedented insights into the electronic structure of elements and materials for any combination of pressure and temperature.

The paper is organized as follows. In section 2, we introduce the relevant theoretical background and the conceptual basis of our work, including the XC kernel and its self-consistent estimation within the framework of KS-DFT, the *static approximation*(56) of the dynamic density response function and the computation of electron–electron correlation functions via the FDT (section 2.3). Section 3 is devoted to the presentation of our new DFT results for the static density response and XC-kernel of both the UEG (section 3.1) and warm dense hydrogen (section 3.2), which, among other things, give us important new insights into the construction of XC functionals for the application in the WDM regime. The paper is concluded by a summary of our main finding, and a discussion of their numerous implications for future works in section 4.

## 2 Theory and Simulation Methods

### 2.1 Linear-Response Theory

Let us consider the following electronic Hamiltonian:^18−20^
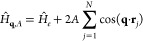
1where the unperturbed system governed by  is subject to a monochromatic external potential of wave vector **q** and amplitude *A*. Clearly, the latter induces a change in the single-electron density, which is given by
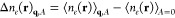
2In the limit of small perturbation amplitudes *A*, the induced density modulation is accurately described by linear-response theory, which gives the relation^57^
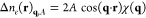
3with χ(**q**) being the static linear response function.

**Figure 2:**
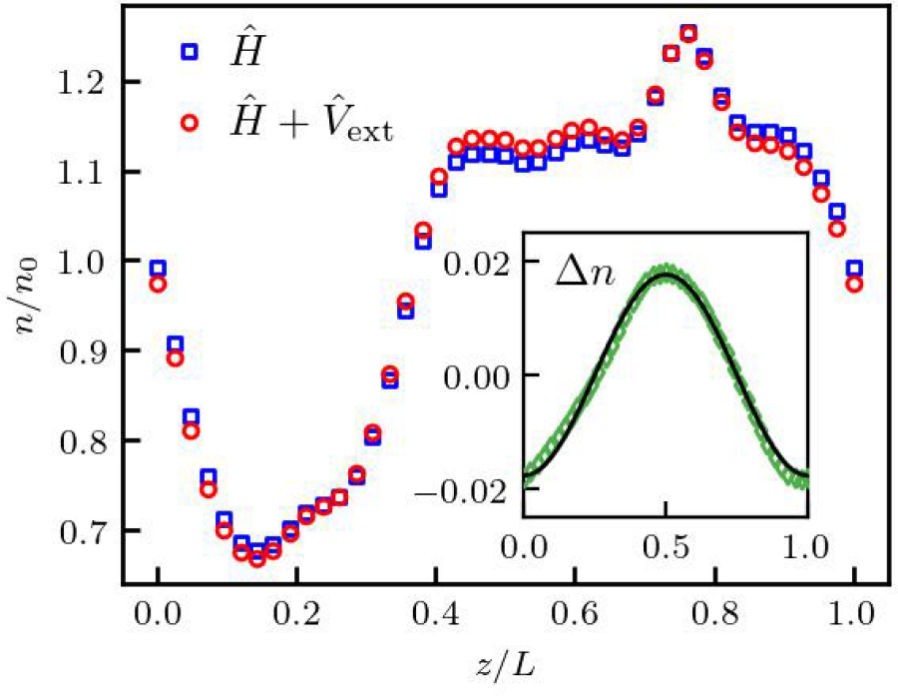
Density response of hydrogen at *r*_*s*_ = 2 and θ = 1, as computed using KS-DFT with meta-GGA level SCAN XC functional. Blue squares show the unperturbed electron density distribution projection along the *z*-axis, i.e., the case without external perturbation (*A* = 0). Red circles are the density perturbation values corresponding to the case with the external harmonic perturbation with *A* = 0.01 along the *z*-axis. The inset shows the density difference Δ*n* (green symbols) between perturbed and unperturbed cases. The solid black curve show the analytical result based on , with χ(*q*) being computed by fitting the DFT data for Δ*n*, according to eq 3.

An example of a density perturbation calculation using the SCAN XC-functional for hydrogen at *r*_s_ =  = 2 (with  being the Wigner–Seitz radius and *a*_B_ is the first Bohr radius^58^) and θ = 1 is presented in Figure 2, where the electron density distribution projections along the *z*-axis are shown for the unperturbed system (blue square) and the perturbed system with *A* = 0.01 and *q* ≃ 0.84*q*_F_ being along the *z*-axis (red circles). The corresponding density difference is shown in the inset, where the resulting density perturbation Δ*n* is given by the green symbols. The density response function is then computed by fitting the Δ*n* data using  based on the least-squares method (solid line), with χ(*q*) being the free parameter.

We note that the presented approach is only exact in the case of homogeneous systems, such as hydrogen averaged over many ion snapshots. Still, the excellent agreement between the simple cosine fit and the induced density in Figure 2 indicates that inhomogeneity effects due to a particular single snapshot are negligible in this regime. In the Appendix, we demonstrate numerically that density perturbation values at higher-harmonics do not contribute to Δ*n*_*e*_ at considered parameters. For completeness, we mention that the presented approach can be further extended for the description of inhomogeneous systems to the microscopic form χ_**G**,**G**′_(**k**, ω), with **G** and **G**′ being the reciprocal lattice vectors^59^ and **k** is a wave vector in the first Brillouin zone; the generalization of our approach to this problem is conceptually straightforward, but not discussed in the present work.

### 2.2 Exchange–Correlation Kernel and Kohn–Sham Response Function

In practice, it is often convenient to express the full dynamic density response function for a homogeneous system as^8,60^
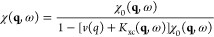
4where χ_0_(**q**, ω) is a known reference function, *v*(*q*) is the Coulomb interaction (*v*(*q*) = 4π/*q*^2^), and *K*_xc_(**q**, ω) is the a priori unknown XC kernel. Generally, only the left-hand side of eq 4 has a well-defined physical meaning as the XC-kernel strongly depends on the particular choice of χ_0_(**q**, ω).^37^ In the case of a uniform electron gas, it is common practice to use the Lindhard function as χ_0_(**q**, ω), which describes the (physical) density response of an ideal Fermi gas at the same parameters. In this case, the XC kernel also has a well-defined physical meaning and contains the full wave-vector- and frequency-resolved information about electronic XC effects in the system. Moreover, it is then directly related to the *local field correction G*(**q**, ω) that is the central property within dielectric theories,^61−66^
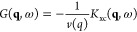
5Hence, setting *K*_xc_ ≡ 0 in eq 4 leads to a description of the electronic density response on the mean-field level, which is commonly known as the *random phase approximation*,^8^
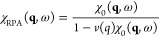
6

In the case of an inhomogeneous electron gas, for example in the potential of a fixed ion configuration, it is common practice to use the KS response function,^59,67^ which is given by
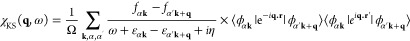
7with ϕ_*n***k**_ and ϵ_α**k**_ being the KS-orbitals and corresponding energy eigenvalues.

In the limit of a UEG, the KS orbitals become plain waves, and eq 7 reverts to the Lindhard function. For nonuniform systems, setting χ_0_(**q**, ω) = χ_KS_(**q**, ω) in eqs 4 and 6 means that the XC kernel constitutes a measure for the deviation between the true density response χ(**q**, ω) and the RPA version of the auxiliary quantity χ_KS_(**q**, ω), cf. eq 8.

In the present work, we present a universal and formally exact strategy to compute the appropriate static XC kernel *K*_xc_(**q**); no external input apart from the usual XC-functional is required.

In particular, we perform KS-DFT simulations to compute the density modulation due to an external monochromatic modulation, cf. eq 2, using an XC-functional of our choice. This gives us results for the static density response function χ(**q**) [eq 3] that are exact on the level of KS-DFT. Having both the physical response χ(**q**) and a reference function such as χ_0_(**q**) = χ_KS_(**q**, 0), it is straightforward to invert eq 4 for the corresponding *static XC kernel*,
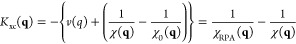
8

Therefore, we can obtain the XC kernel using χ(*q*) and χ_KS_(*q*) (χ_RPA_(*q*)) without computing the second-order functional derivatives explicitly.

### 2.3 Static Approximation and Fluctuation–Dissipation Theorem

The main present limitation of our new approach is given by its restriction to compute the XC kernel in the limit of ω = 0. Still, it is possible to compute the dynamic density response function within the *static approximation*,^56^
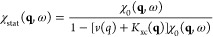
9where the dynamic XC kernel *K*_xc_(**q**, ω) is approximated by its exact static limit. Equation 9 thus combines a dynamic description on the level of the RPA with exact static correlations. This approximation has been shown to be highly accurate in the case of the UEG for weak to moderate coupling strengths, including the particularly relevant regime of metallic densities *r*_s_ ≲ 5.^56^

The fluctuation–dissipation theorem^8^ then gives a straightforward relationship between the dynamic density response function χ(**q**, ω), and the dynamic structure factor *S*_ee_(**q**, ω):
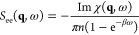
10

The DSF constitutes the key property in state-of-the-art XRTS experiments.^68,69^ Therefore, eqs 9 and 10 open up the possibility to compare KS-DFT simulation results that have been obtained via *K*_xc_(**q**) and χ_0_(**q**, ω) to an experimental measurement.

In addition, eq 10 provides straightforward access to the static structure factor,
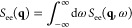
11i.e., the Fourier transform of the usual pair correlation function *g*_ee_(**r**). In combination, eqs 9–11 imply that one can use KS-DFT to compute electron–electron correlation functions without any additional external input. The interaction energy *W* then follows from an additional integration over the wave vector **q**. Finally, one might utilize the well-known adiabatic connection formula^8^ (resulting in an integration over an effective coupling parameter λ ∈ [0, 1]) to obtain the free energy, which contains the full thermodynamic information about the system of interest.

## 3 Results: Static Density Response and XC Kernel

### 3.1 Uniform Electron Gas

**Figure 3:**
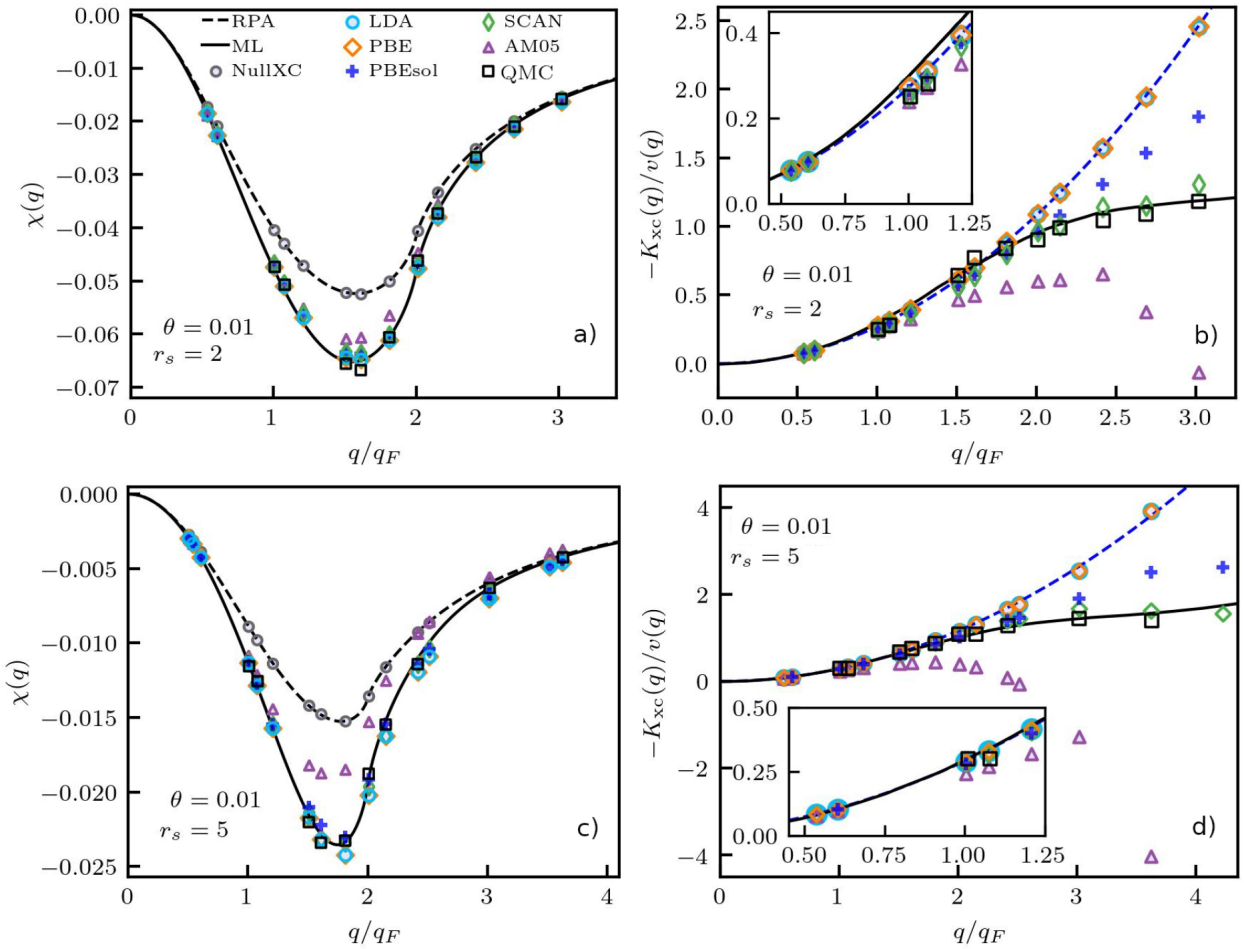
Electronic static density response function χ(**q**) (left column) and XC kernel *K*_xc_(**q**) (right column) of the UEG under ambient conditions (Θ = 0.01) for *r*_s_ = 2 (top row) and *r*_s_ = 5 (bottom row). Solid (dashed) black line: exact UEG results based on the neural-net representation of ref (70) (analytical RPA). Black squares: exact QMC results by Moroni et al.^19^ The other symbols distinguish DFT calculations for the density modulation described by eq 2 using different XC functionals; see panel (a) and the main text.

Let us begin our investigation of electron–electron correlation functions based on density functional theory (DFT) and the fluctuation–dissipation theorem with an analysis of the static density response function χ(**q**) of the UEG under ambient conditions (i.e., at *T* = 0) shown in Figure 3. In particular, we have performed DFT calculations governed by the perturbed Hamiltonian  [cf. eq 1] for multiple wave vectors **q** and a sufficiently small perturbation amplitude *A*; the different symbols show results for a selection of widely used XC functionals. As a reference, we also include the exact response of the UEG as the solid black lines, which are based on QMC results by Moroni et al.^19^ (black squares) and are taken from the neural-net representation from ref (70).

Figure 3a has been obtained for *r*_s_ = 2, which is a metallic density that can be probed in experiments, for example, with aluminum.^71^ Evidently, all curves exhibit the same qualitative trends, i.e., the exact limit of perfect screening,^72^
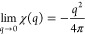
12and the noninteracting limit
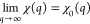
13for small and large wave numbers *q* = |**q**|, respectively. For *q* ≲ *q*_F_ (with  being the Fermi wavenumber^8^), all DFT curves using a nonzero XC-functional are in excellent agreement to the exact results. This can be seen particularly well in panel (b), which shows the corresponding deviations to the mean-field curve (eq 8); see the discussion below. The good agreement is a direct consequence of the well-known compressibility sum-rule, see eq 14 and the corresponding discussion below. The most pronounced differences between the different functionals occur for intermediate wave numbers 1.5*q*_F_ ≲ *q* ≲ 2.5*q*_F_, where χ(**q**) exhibits a negative peak. From a physical perspective, this feature can be explained by the spontaneous alignment of electron pairs,^73^ which leads to a reduction in the free-energy landscape and, therefore, an increased density response. This pair alignment is highly sensitive to electronic XC effects, which leads to the observed impact of the XC functional. For larger *q*, the impact of the XC kernel again decreases, although some deviations remain over the entire depicted *q*-range.

Let us next analyze the respective accuracy of the various XC functionals. First, we note that the KS-response function (cf. eq 7), when being inserted into the RPA expression described by eq 6, reproduces the analytical RPA (dashed black curve) for all functionals, as the respective KS-orbitals of the unperturbed UEG are always plain waves. Similarly, evaluating the density modulation eq 2 based on a harmonically perturbed DFT simulation and setting *E*_xc_[*n*_*e*_] ≡ 0 (gray circles) gives the same mean-field description. Regarding the different approximations for *E*_xc_[*n*_*e*_], we find that both the LDA functional by Perdew and Wang^74^ (light blue circles) and the generalized gradient approximation (GGA) by Perdew, Burke, and Ernzerhof (PBE,^75^ orange diamonds) give indistinguishable results. This is expected as all gradient terms vanish in the case of a UEG. The comparison to the exact QMC data gives good qualitative agreement for *q* ≲ 2*q*_F_, and deterioration in the quality for larger wave numbers *q*. Let us next consider the AM05 functional by Armiento and Mattson^76^ (purple up-triangles), which is a semilocal GGA, and has been shown to give comparable quality to hybrid functionals in the description of solids.^77^ Moreover, it has been applied to the calculation of electronic structures at WDM parameters.^78−80^ Here, we find that AM05 is the least accurate functional and substantially underestimates the true depth of the minimum in the static density response. The semiempirical PBEsol^81^ (blue plus signs) constitutes a significant improvement over PBE for *q* ≳ 2*q*_F_. Finally, the meta-GGA SCAN^82^ (green diamonds) exhibit the best performance, as expected.

To get a more rigorous insight into the performance of the different functionals, we show the corresponding XC kernel *K*_xc_(**q**) that we have obtained by evaluating eq 8 in Figure 3b). Throughout this work, we follow the usual convention^70,83,84^ and divide *K*_xc_ by the Coulomb interaction *v*(*q*), resulting in the commonly analyzed local field correction, cf. eq 5. In the limit of small *q*, the LFC is know to satisfy the exact compressibility sum-rule,^83^
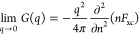
14with *n* = *N*/*V* being the average number density. It is depicted as the dashed blue parabola in Figure 3; we note that it holds lim_*T*→0_*F*_xc_ = *E*_xc_ in the ground-state limit. Evidently, eq 14 is accurately reproduced both by the exact neural-net representation and by all depicted XC-functionals in the limit of small *q*. Moreover, both the LDA and PBE functionals have been constructed to reproduce eq 14 for all *q*(85) in the case of the UEG, which is substantiated by the presented empirical analysis. Remarkably, the parabolic small-*q* expansion remains reasonably accurate for *q* ≲ 2*q*_F_; this is a highly nontrivial observation and explains the success of both the simple LDA and the somewhat more sophisticated PBE in the description of bulk materials that has been reported in previous investigations.^19^

In contrast, the AM05 functional only reproduces eq 14 for *q* ≲ 1.2*q*_F_. For large *q*, it has been designed to reproduce the Airy gas model,^77^ resulting in a substantial, unphysical drop toward negative values in this regime. The semiempirical PBEsol, on the other hand, is virtually indistinguishable from PBE for *q* ≲ 2.5*q*_F_, and exhibits a somewhat higher accuracy at large wavenumbers. Finally, SCAN constitutes, by far, the most accurate functional and gives basically exact results over the entire depicted *q*-range, since it was designed to reproduce the exact ground-state QMC results in this range of wavenumbers.^82^

In the bottom row of Figure 3, we show the same analysis for a lower density (*r*_s_ = 5). Physically, this is located near densities of the conduction electrons in Potassium and Rubidium, and can also be probed in evaporation experiments such as hydrogen jets.^86^ Due to the role of *r*_*s*_ as the *quantum coupling parameter*,^8,29^ the electrons are more strongly correlated at these conditions, which results in a sharper minimum in χ(**q**). Consequently, the impact of the different XC-functionals is more pronounced. Overall, we find the same qualitative trends as for *r*_*s*_ = 2: AM05 is, by far, the least accurate functional, whereas SCAN is virtually exact for all depicted *q*; eq 14 is accurate for *q* ≲ 2*q*_F_, which explains the high accuracy of LDA, PBE, and PBEsol; the latter constitutes a substantial improvement over PBE for *q* ≳ 3*q*_F_.

**Figure 4:**
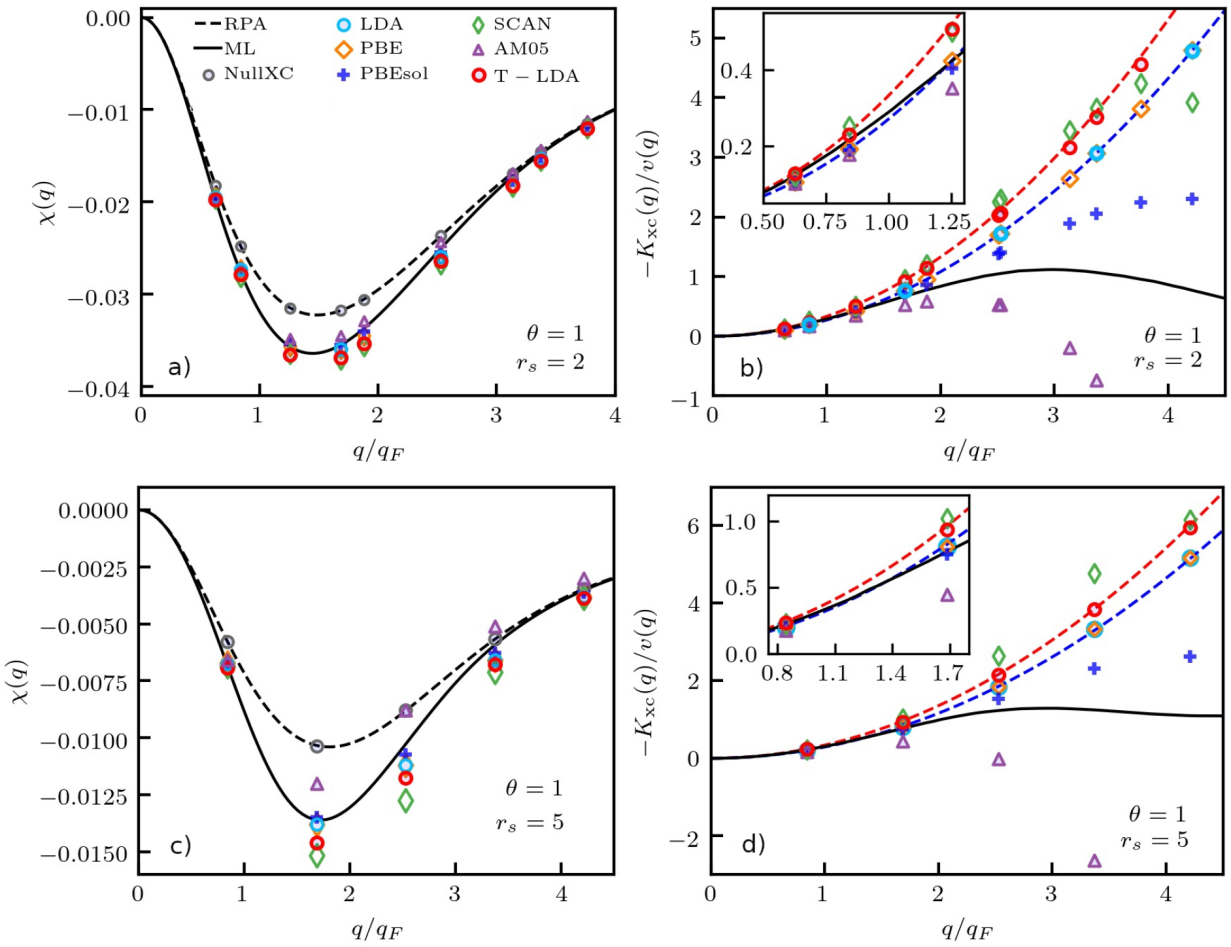
Electronic static density response function χ(**q**) (left column) and XC kernel *K*_xc_(**q**) (right column) of the UEG under WDM^45,46^ conditions (Θ = 1) for *r*_s_ = 2 (top row) and *r*_s_ = 5 (bottom row). Solid (dashed) black line represents exact UEG results based on the neural-net representation of ref (70) (analytical RPA). The other symbols distinguish DFT calculations for the density modulation (eq 2) using different XC functionals; see panel (a) and the main text.

Let us next repeat this analysis for the UEG in the WDM regime, i.e., at the electronic Fermi temperature Θ = 1 shown in Figure 4. First, we note that, due to the increased temperature, the impact of Coulomb correlation effects is decreased. Consequently, the negative minimum in χ(**q**) is less pronounced compared to *T* = 0 for both values of *r*_s_. At the same time, we find substantially more pronounced differences between the various depicted XC functionals. In particular, the finite-*T* LDA functional by Groth et al.^51^ (red circles) is even somewhat less accurate, compared to the ground-state LDA and PBE.

To understand these counterintuitive observations, we must consider the XC kernel shown in the right column of Figure 4. In particular, the dashed red and blue lines show the exact small-*q* expansion eq 14 evaluated at Θ = 1 and Θ = 0, respectively. Evidently, the ground-state LDA (and PBE) follows the latter curve, as it is expected. Similarly, the finite-*T* LDA follows the red curve and, therefore, reproduces the correct impact of the temperature on the small-*q* limit. This can be seen particularly well in the inset, where we show a magnified segment. It can be expected that even the recent finite-*T* extension of PBE by Karasiev et al.^52^ exhibits the same behavior as it has been constructed to reproduce the finite-*T* LDA. In practice, however, the impact of *K*_xc_(**q**) on the density response function vanishes for *q* → 0, and even the RPA (eq 6) becomes exact. For *q* ≳ *q*_F_, where the impact of the XC kernel is most pronounced, the ground-state evaluation of eq 14 constitutes a superior approximation to the true curve (solid black). Therefore, the ground-state LDA exhibits a superior accuracy compared to the theoretically more consistent temperature-dependent functional.

**Figure 5:**
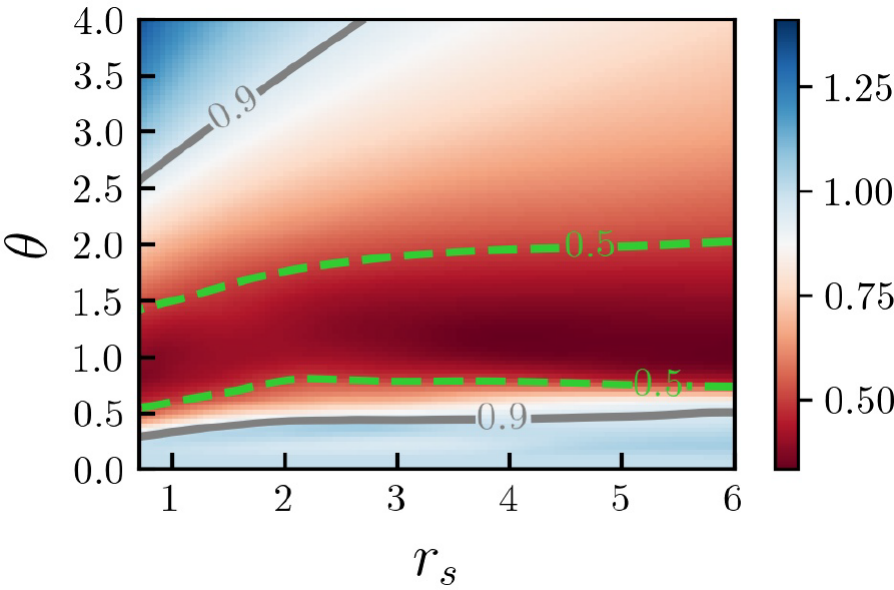
Color-map illustrating parameters where ground-state LDA works better than T-LDA. See eq 15 and corresponding discussion in the main text.

This is a highly important point that deserves a more detailed investigation. To this end, we define a *relative agreement measure* (RAM) between the LFCs evaluated from the ground-state and finite-*T* LDA functionals toward the true LFC of the UEG as
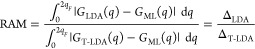
15Here, *G*(*q*) corresponds to the local field correction defined in eq 5 above. We note that the upper limit of 2*q*_F_ has been chosen based on the empirical approximate validity range of the small-*q* expansion (eq 14); using larger upper limits always favors the ground-state LDA, which has a tendency to have a smaller prefactor in the parabolic expansion. The results for eq 15 are shown as the heat-map in Figure 5 in the *r*_s_-θ-plain covering the entire range of metallic densities and the temperatures that are most relevant for WDM research.^29,45,46^ In particular, a RAM value of <1 signifies that the ground-state LDA provides an overall more-accurate kernel, compared to the finite-*T* functional. At Θ = 0, both LDA representations are, by construction,^51^ identical. Remarkably, the ground-state LDA becomes substantially more accurate at Θ ≳ 0.3, and the lowest RAM values are obtained between the two dashed green lines, i.e., at Θ ≈ 1. This is precisely the regime where the impact of the temperature on the XC functional has the most influence on the total free energy *F*[*n*_*e*_]^48,49^ and, therefore, on the outcome of a DFT simulation. Yet, as we have seen above, this leads to a larger prefactor in eq 14 and, therefore, a less-accurate XC kernel for *q* ≳ 0.5*q*_F_ on the level of the LDA. For completeness, we note that the RAM value only exceeds unity for *r*_s_ ≈ 1 and θ ≈ 4 in the presented overview; the impact of *K*_xc_, however, is negligible, due to the high temperature and density.

We stress that these findings have profound consequences for the construction of the next generation of XC functionals that are specifically designed for the application under WDM conditions. Evidently, translating a Jacob’s ladder of functional approximations^4^ from the ground-state to finite temperatures does not necessarily improve the quality of DFT simulations in the WDM regime. Making the lowest rung—i.e., the LDA—explicitly *T*-dependent might actually lead to a deterioration of the attained accuracy. Moreover, this deficiency is, by design, not removed on the GGA level, which is based on the same *q* → 0 expansion. Returning to Figure 4, we further find that the ground-state SCAN functional performs similarly poorly as AM05, which is in stark contrast to its impressive accuracy at *T* = 0 (cf. Figure 3). We thus conclude that the meta-GGA corrections on which SCAN is based are strongly dependent on the electronic temperature implicitly. A temperature correction to SCAN computed on the GGA level as it has recently been proposed in ref (87) would likely only increase the systematic errors in the present case.

Our analysis of the density response and XC kernel of the UEG leads to a recommendation for the construction of novel XC functionals that fulfill the demanding requirements of WDM theory. As we have seen, it is important to construct a functional that combines the correct *T* dependence of eq 14 with an accurate description of XC effects over the entire relevant *q*-range. This is particularly important for WDM applications, where large *q* play a more important role in practical applications, compared to ambient conditions.^45^ In this regard, a promising candidate is given by a new class of nonlocal functionals based on the adiabatic connection formula and the fluctuation–dissipation theorem.^88,89^

### 3.2 Warm Dense Hydrogen

**Figure 6:**
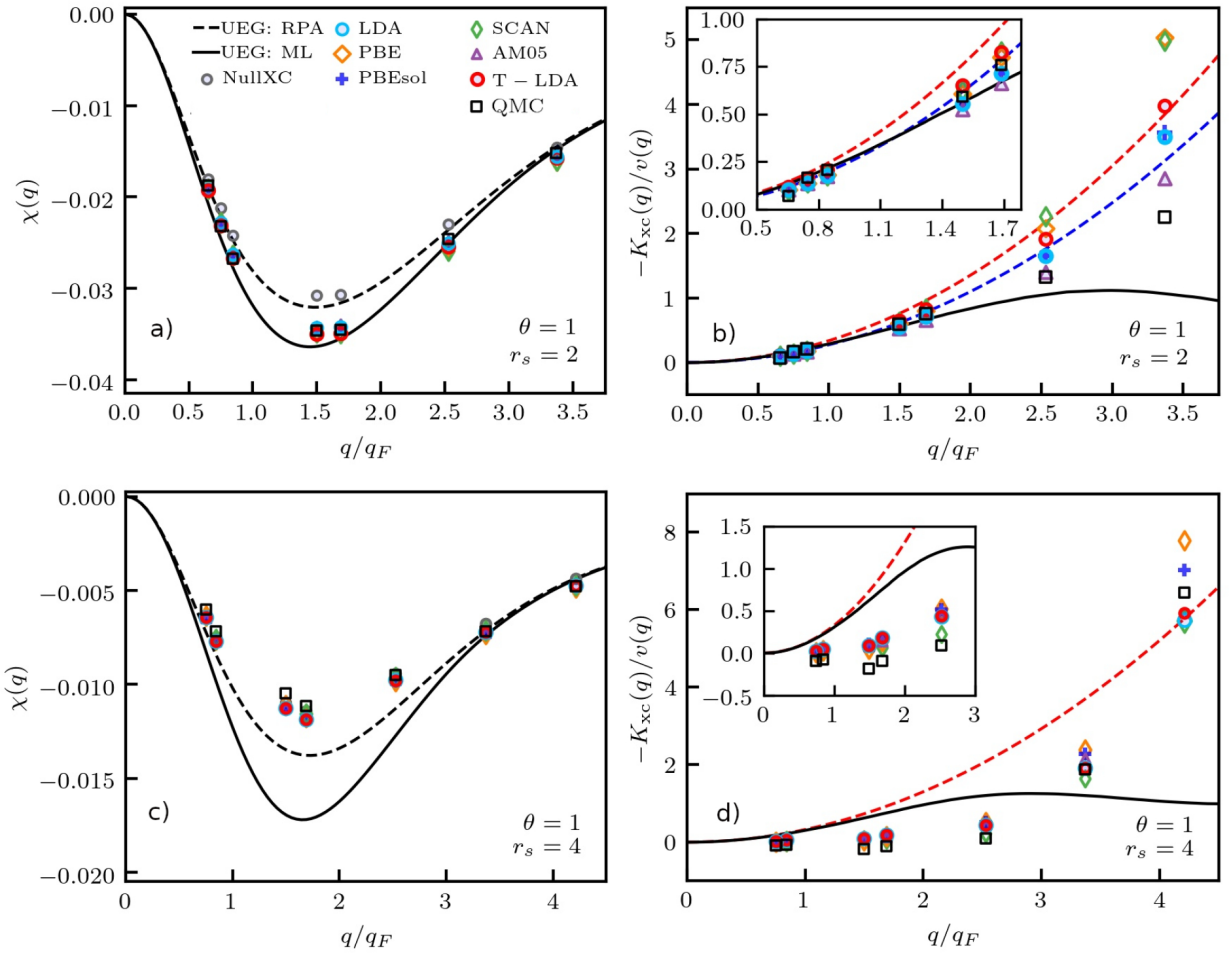
Electronic static density response function χ(**q**) (left column) and XC-kernel *K*_xc_(**q**) (right column) computed from the same mean-field reference function (see the main text) χ_0_(**q**) of hydrogen under WDM^45,46^ conditions (Θ = 1) for *r*_s_ = 2 (top row) and *r*_s_ = 4 (bottom row). Solid (dashed) black line represents exact results for the UEG model under the same conditions based on the neural-net representation of ref (70) (analytical RPA). Black squares represent exact QMC results for hydrogen by Böhme et al.^37^ The other symbols distinguish DFT calculations for the density modulation (eq 2) using different XC functionals; see panel (a) and the main text.

To demonstrate the broad utility of our new approach, we next consider hydrogen under extreme conditions—a state of matter that plays a central role in the description of the implosion path of a fuel capsule toward nuclear fusion^41^ and naturally occurs within a gamut of astrophysical objects, such as giant planet interiors.^39^ In Figure 6, we show our new DFT results for the static density response of hydrogen that has been computed for a single fixed configuration of proton coordinates, i.e., a single ion snapshot from a corresponding DFT-MD simulation. We note that, while the averaging over many snapshots is straightforward, benchmarking DFT for a single proton configuration constitutes an even more rigorous test of our methodology as, in this way, error cancellation between different snapshots is ruled out. The left column of Figure 6 shows results for χ(**q**) at Θ = 1, and the top and bottom rows have been obtained for *r*_s_ = 2 and *r*_s_ = 4. For these parameters, we are able to compare our new DFT results to exact QMC data by Böhme et al.^37^ (black squares) that have been obtained for the same ion configuration. In addition, we also include both the exact (solid black) and RPA results (dashed black) for the UEG model under the same conditions.^70^

At the higher density, where hydrogen is known to be mostly ionized, the bulk of the electrons can be categorized as *unbound*, meaning that they are not primarily localized around the protons. Therefore, the density response of hydrogen closely resembles the UEG model under these conditions. In addition, we find that our DFT evaluation of the perturbed density eq 2 is in good agreement with the QMC reference data for all *q*, and is only weakly dependent on the functional employed. In particular, the difference between ground-state (light blue circles) and finite-*T* (red circles) LDA is small, with the former being a trifle more accurate. The corresponding results for the static XC kernel are shown in the right column of Figure 6 and, overall, closely resemble our earlier findings for the UEG model (cf. Figure 4 above).

From a physical perspective, the case of *r*_s_ = 4, shown in Figure 6c, is even more interesting. In addition to the more pronounced impact of Coulomb correlations (and, therefore, electronic XC effects), hydrogen is partly ionized under these conditions, with an approximate fraction of *free electrons* of α = 0.54–0.6.^37,90^ Consequently, the numerical results for χ(**q**) exhibit a substantially reduced density response,(the compared to the UEG model, as the *bound* electrons cannot react to the external potential. Overall, we find good qualitative agreement between DFT and the QMC data over the entire depicted *q*-range, even though the true reduction of the density response due to the localization around the protons is somewhat underestimated around the vicinity of the negative minimum, i.e., *q*_F_ ≲ *q* ≲ 3*q*_F_. Remarkably, we find that all XC functionals reproduce the nontrivial increase in the magnitude of χ(**q**), compared to the ,UEG model around *q* ∼ 4*q*_F_, which has very recently been explained as a consequence of isotropy breaking in the presence of the proton configuration in ref (37).

In Figures 6b and 6d, we show the corresponding XC kernels that we have extracted from the different χ(**q**) datasets (both QMC and DFT evaluations of eq 2 using different XC functionals) via eq 8, but using the same data for the reference function χ_0_(**q**) that we have obtained from a separate DFT simulation with the XC functional being set to zero. This has the advantage that XC kernels from different theories are directly comparable to each other. For completeness, we note that extracting the actual XC-functional-dependent kernel by inserting the respective χ_KS_(**q**, 0) into eq 8 is straightforward, but would make the direct comparison less meaningful. The resulting data for *K*_xc_(**q**) of hydrogen at *r*_s_ = 4 and Θ = 1 qualitatively agree with each other, but starkly disagree from the UEG model under these conditions. In particular, we do not find the simple parabolic behavior observed in eq 14 for LDA/PBE. In addition, the kernel obtains remarkably small values for *q* ≲ 2.5*q*_F_, followed by a pronounced increase for *q* ≳ 3*q*_F_. Clearly, our new methodology is capable to accurately capture the complex interplay of the ion structure with electronic XC effects as they manifest in *K*_xc_(**q**).

To understand the observed differences of the actual kernel of hydrogen to the UEG model, we must go back to Figure 6c, where we show the mean-field results (corresponding to χ_RPA_(**q**)) as the gray circles. Interestingly, these data are in excellent agreement to the other datasets. In fact, the very small values of *K*_xc_(**q**) that have been obtained by inserting the QMC data into eq 8 directly indicate that the results that have been obtained without an XC functional are more accurate than the other DFT data. This is, however, likely coincidental and comes as a result of the crossover from the UEG-like behavior of hydrogen at Θ = 1 for *r*_s_ ≲ 2 to the case of atomic/molecular hydrogen at large *r*_s_, where the electrons are predominantly localized around the protons. In the former limit, it is well-known that the RPA underestimates the true density response,^29^ whereas, in the latter case, it underestimates the true degree of localization around the ions, resulting in an effective overestimation of the magnitude of χ(**q**). The present case of *r*_s_ = 4 is located between these two limits, and the apparent accurate description of χ(**q**) by the mean-field calculation is a direct consequence of the cancellation of RPA errors, which have a positive (negative) sign for small (large) *r*_s_; other observables such as the single-electron density *n*_e_(**r**) are less accurately reproduced by the mean-field calculations, compared to the other depicted XC functionals.

The observed stark increase in *K*_xc_(**q**) for large wave numbers also can be directly traced back to the behavior of χ_0_(**q**) and the depicted mean-field response χ_RPA_(**q**), which does *not* reproduce the increase in magnitude of the density response, compared to the UEG model under these conditions. Consequently, the latter predominantly constitutes an XC effect that is determined by the XC kernel, and, therefore, is accurately captured by our new methodology.

**Figure 7:**
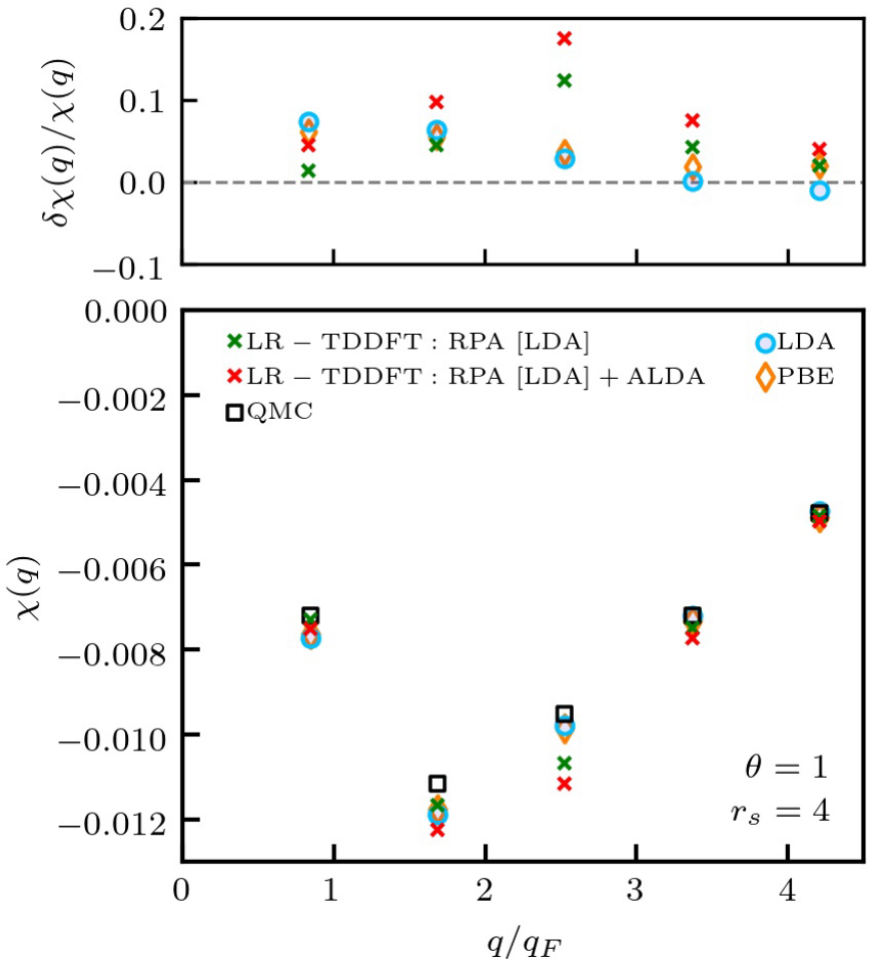
Illustration of the inconsistent combination of χ_KS_(**q**, ω) with the ALDA kernel. (Bottom) Static density response of warm dense hydrogen with *r*_*s*_ = 4 and θ = 1; the light blue circles, orange diamonds, and black squares are taken from Figure 6. Green crosses represent the static (ω → 0) limit of LR-TDDFT results within RPA (eq 6) based on χ_KS_(**q**, ω) using KS-orbitals from a ground-state LDA calculation. Red crosses denote corresponding ALDA results. (Top) Relative error with respect to exact QMC benchmark data. The inconsistent incorporation of the ALDA model kernel leads to a deterioration, compared to RPA, for all depicted wave numbers *q*.

Let us conclude this analysis of the static density response of warm dense hydrogen by comparing our new approach to the current state of the art. In Figure 7, we again consider hydrogen at *r*_s_ = 4 and Θ = 1, and the black squares, blue circles, and orange triangles respectively show the QMC, LDA, and PBE results from Figure 6. In addition, the green crosses have been obtained following the standard paradigm within LR-TDDFT, that is, computing the reference function χ_0_(**q**, ω) in the limit of ω → 0 on the basis of the KS orbitals (cf. eq 7) from a DFT simulation of the unperturbed system using the LDA functional. Both the KS-response function and the corresponding RPA are defacto uncontrolled approximations. In practice, the green crosses are accurate for small *q*, but lead to substantial deterioration in the accuracy for *q* ≳ 2*q*_F_, compared to the LDA evaluation of eq 2 proposed in the present work; this can be seen particularly well in the top panel showing the relative deviation from the exact QMC reference data. Even worse, including the widely used ALDA model as the XC kernel (red crosses)—a standard practice within LR-TDDFT^59,67^—*actually increases the systematic errors* for all *q*. This constitutes an unambiguous demonstration of the practical impact of the inconsistent combination of a KS-response function with an XC kernel from a different model, which is overcome by the approach presented in this work.

## 4 Conclusions and Outlook

We have presented a formally exact framework to compute the electronic static XC kernel within KS-DFT without any additional external input apart from the usual XC functional. Our methodology provides access to the static XC kernel across all rungs of Jacob’s ladder, including promising hybrid functionals.^91^

As the first application, we have studied the UEG model, which is the archetypical system of interacting electrons and plays a central role in the context of DFT. Under ambient conditions (i.e., *T* = 0), DFT simulations of the harmonically perturbed electron gas accurately reproduce the static linear density response function χ(**q**) over the entire *q*-range. This is a direct consequence of the small-*q* limit of the XC kernel (eq 14), which, remarkably, reproduces the exact static kernel for *q* ≲ 2*q*_F_.^19^ Regarding *K*_xc_(**q**) itself, we have found that SCAN constitutes by far the most accurate functional at *T* = 0 and is basically exact for all *q*.

An additional interesting research question is the performance of different XC functionals in the WDM regime, i.e., at Θ = 1. Interestingly, we have found that the ground-state LDA/PBE functionals perform better than their consistently temperature-dependent counterparts. Our analysis has revealed that this is a nontrivial consequence of the compressibility sum-rule described by eq 14: the *T*-dependent LDA is indeed superior in the limit of *q* → 0, but, here, the impact of *K*_xc_(**q**) on the actual density response and related properties is negligible. For *q* ≈ *q*_F_, the ground-state expansion more accurately reproduces the true XC kernel, which results in a superior accuracy of *T* = 0 LDA and GGA functionals at Θ = 1. This insight has profound consequences for the development of the next generation of XC-functionals that are specifically designed for the application under WDM conditions.

As the next step, we have performed a similar analysis for warm dense hydrogen, which is of prime importance for technological applications such as nuclear fusion and a host of astrophysical applications. Overall, we have found that the DFT evaluation of the density modulation due to an external perturbation (eq 2) is indeed capable to very accurately, though not exactly, describe the density response of hydrogen both for *r*_s_ = 2 and *r*_s_ = 4. In particular, our method captures the nontrivial increase in magnitude of the density response for *q* ≳ 3*q*_F_ due to the isotropy breaking of the proton configuration;^37^ we stress that this feature is decisively shaped by electronic XC effects and, therefore, is not reproduced on the level of the RPA. In other words, the accurate, system-specific XC kernel is indispensable. For *r*_s_ = 2, hydrogen is predominantly ionized and *K*_xc_(**q**) exhibits an UEG-like behavior. Yet, the UEG model breaks completely down at *r*_s_ = 4 as the physical behavior of the system is substantially shaped by the localization of a substantial fraction of the electrons around the protons. Our new approach, on the other hand, accurately captures the actual behavior of the XC kernel known from exact QMC simulations^37^ over the entire *q*-range, with both the ground-state and finite-*T* LDA functionals being the most accurate.

Many research fields within physics, chemistry, material science, and related disciplines will benefit from the framework that has been demonstrated in this work. First and foremost, we note that the XC kernel is the key ingredient to a host of practical applications, such as LR-TDDFT, the construction of electronically screened effective potentials,^6−8^ the incorporation of correlation effects into quantum hydrodynamics,^9−11^ plasmonics,^92^ and the estimation of the energy loss characteristics of high-energy density plasmas.^12−14^ A particularly important example is given by the interpretation of XRTS experiments within the widely used Chihara approximation,^21,93^ which gives one direct access to electronic DSF to such system parameters as the electronic temperature and density.

The presented idea is not limited to electronic pair correlations. A gradual increase in the amplitude *A* of the external harmonic perturbation gives one straightforward access to the *nonlinear electronic density response*(20,57,94) of any given system, which, in turn, is directly connected to higher-order correlation functions between three and more electrons.^95^ In other words, the presented approach can give one access to the full hierarchy of many-electron correlations within the framework of DFT, and without any additional external input.

On the one hand, our methodology will directly benefit from the availability of more sophisticated XC functionals on higher rungs of Jacob’s ladder such as the promising hybrid functional by Heyd, Scuseria, and Ernzerhof (HSE) .^91^ On the other hand, the analysis of the XC kernel *K*_xc_(**q**) on the basis of a particular functional can give valuable insights to guide new developments, as we have demonstrated for the case of WDM.

## Figures and Tables

**Figure A1:**
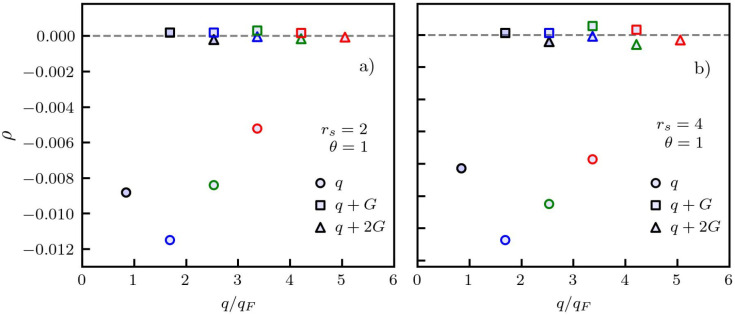
Contributions to the total density change from density perturbation values at different wave numbers for a single snapshot in warm dense hydrogen at (a) *r*_s_ = 2, and (b) *r*_s_ = 4 for θ = 1. The perturbation amplitude in eq 1 is set to *A* = 0.01. Circles show the density perturbation value at the same wavenumber *q* as of the external perturbation. Squares and triangles show the density perturbation values at *q* + *G* and *q* + 2*G*, respectively, induced by the external field with the wavenumber *q*. Here, *G* is the reciprocal lattice vector (*G* = 2π/*L*). Different colors represent data for different *q* values. The data are presented in units of mean density (*n*_0_ = *N*/*L*^3^).

## Data Availability

The data supporting the findings of this study are available on the Rossendorf Data Repository (RODARE).^96^

## Appendix

### Simulation Details

For the KS-DFT calculations of the presented XC kernels and dynamic response functions within LR-TDDFT, we used the GPAW code,^97−100^ which is a real-space implementation of the projector augmented-wave method. For the calculation of the XC kernel presented in Figures 3, 4, and 6, the following parameters have been used: For the UEG at θ = 0.01, *r*_s_ = 2 and *r*_s_ = 5, the calculations were performed with 38, 54, and 66 particles in the main cell. For the UEG at θ = 1 and *r*_s_ = 2, the simulations were performed with 14 and 34 particles. This is consistent with previous QMC investigations,^20,70,101,102^ where it has been shown that finite-size errors are negligible at the present conditions. For the UEG at θ = 1 and *r*_s_ = 5, the calculations were performed with 14 particles. For hydrogen at θ = 1 and *r*_s_ = 2, the simulations were performed with 14, 20, and 30 particles in the main cell. For hydrogen at θ = 1 and *r*_s_ = 4, the calculations were performed with 14 and 20 particles.

The main cubic cell size is computed as *L* = . Accordingly, perturbation wave numbers (set along the *z*-axis) are defined by *L* as *q* = η × 2π/*L*, with η being a positive integer number. We used a Monkhorst–Pack^103^ sampling of the Brillouin zone. For the UEG at *r*_s_ = 2 (*r*_s_ = 5), a *k*-point grid of *N*_*k*_ × *N*_*k*_ × *N*_*k*_ total points with *N*_*k*_ = 12 (*N*_*k*_ = 8) was used. For hydrogen, we used *N*_*k*_ = 8. The cutoff energy was set to 800 eV at θ = 1 and *r*_s_ = 2, and to 440 eV at other *r*_s_ and θ values. The number of bands in the case of the UEG at *r*_s_ = 2 and θ = 1 was set to *N*_b_ = 500 (with the smallest occupation number *f*_min_ ≲ 10^–7^). At *r*_s_ = 5 and θ = 1, we used *N*_b_ = 240 bands with *f*_min_ ≲ 10^–6^. For the UEG at θ = 0.01, we used *N*_b_ = 70 bands for *N* = 66 particles, and *N*_b_ = 2*N* for *N* = 20 and *N* = 14 particles. For hydrogen at *r*_s_ = 2 and θ = 1, the number of bands was set to *N*_b_ = 440 (for 14 and 20 particles) and *N*_b_ = 600 (for 30 particles). For hydrogen at *r*_s_ = 4 and θ = 1, the number of bands was set to *N*_b_ = 300 (for 14 particles) and *N*_b_ = 400 (for 20 particles).

For the UEG at *r*_s_ = 2 (*r*_s_ = 5), the perturbation amplitude was set to *A* = 0.01 (*A* = 0.002), with *A* being in Hartree atomic units. For hydrogen at *r*_*s*_ = 2 and *r*_*s*_ = 4, the perturbation amplitude was set to *A* = 0.01.

The simulation results were cross-checked by performing UEG calculations using Abinit^104−107^ for LDA, PBE, and PBEsol XC functionals at the same parameters.

For the calculation of the static KS density response function of hydrogen at *r*_s_ = 4 and θ = 1, the main simulation cell size was set to *L* = 8.224 (with 14 particles in the main simulation cell), the number of bands 1900, the *k*-point grid 4 × 4 × 4, and the cut-off energy in the thermal equilibrium calculation was set to *E*_cut_ = 400 eV. For the computation of the static KS density response function a plain-wave cut-off of 90 eV was used and the wave numbers *q* = *jq*_min_, with *j* = 1, ..., 5 (*j* = 1, ..., 4), were considered.

### Full Wavenumber Dependence of the Density Perturbation

In Figure A1, we show contributions to the total density change from density perturbation values at different wave numbers for a single snapshot. Particularly, we demonstrate that density perturbation values at *q* + *G* and *q* + 2*G* can be safely neglected, where *G* = 2π/*L*. Calculations are performed for warm dense hydrogen at *r*_*s*_ = 2 and *r*_*s*_ = 4. We consider partially degenerate case with θ = 1. The results are computed by analyzing the density perturbation components in Fourier space. The results in Figure A1 are for the case with the LDA functional.
